# APACHE II, SOFA, and SAPS III after transhiatal and thoracoscopic in prone position esophagectomy for esophageal cancer: a single-center retrospective cohort analysis

**DOI:** 10.1590/0102-67202025000028e1897

**Published:** 2025-09-29

**Authors:** José Antonio Possatto FERRER, Valdir TERCIOTI, Antonio Luis Eiras FALCÃO, João de Souza COELHO, Ary Augusto de Castro MACEDO, Nelson Adami ANDREOLLO, Luiz Roberto LOPES

**Affiliations:** 1 Universidade Estadual de Campinas, University Hospital, Faculty of Medical Sciences and Gastrocentro, Digestive Diseases Surgical Unit, Department of Surgery – Campinas (SP), Brazil.; 2 Universidade Estadual de Campinas, University Hospital, Faculty of Medical Sciences, Intensive Care Unit, Department of Surgery – Campinas (SP), Brazil.

**Keywords:** Esophageal Neoplasms, Esophagectomy, Thoracoscopy, Critical Cares, Prognosis, Minimally Invasive Surgical Procedures, Neoplasias Esofágicas, Esofagectomia, Toracoscopia, Cuidados Críticos, Prognóstico, Procedimentos Cirúrgicos Minimamente Invasivos

## Abstract

**Background:**

Esophagectomy is a major, invasive, and long-lasting surgery performed in patients with comorbidities and compromised nutritional conditions. The historical challenges of surgical treatment of esophageal cancer are to overcome mortality, improve survival, and decrease morbidity.

**Aims:**

The aim of the study is to compare the intraoperative morbidity of two distinct surgical techniques of esophagectomy in esophageal cancer, transhiatal esophagectomy and video-assisted thoracoscopy in the prone position, analyzing intraoperative physiological parameters, scores on admission to the intensive care unit (ICU) (APACHE II, SOFA, and SAPS III), and postoperative evolution.

**Methods:**

Retrospective, cross-sectional study evaluating patients admitted to the ICU in the immediate postoperative period of elective esophagectomy for esophageal neoplasia (squamous cell carcinoma and adenocarcinoma). Data were obtained from a computerized registry database of the ICU and from patient records.

**Results:**

Sixty-three patients over 18 years of age were evaluated and divided into two groups: 31 (49.21%) underwent transhiatal esophagectomy, and 32 (50.79%) underwent videoassisted thoracoscopic esophagectomy. No statistically significant difference was observed for length of ICU stay (p=0.5309), length of postoperative hospital stay (p=0.3066), or death in the perioperative period (30 days, p=0.6562). Regarding intraoperative parameters, no statistically significant difference was observed for patients who received blood transfusion (p=0.2097); amount in milliliters (p=0.2893); patients who used vasoactive drugs (VADs) (p=0.9243); time VAD use (p=0.9327); volume of fluids infused in milliliters (p=0.7825); or diuresis in milliliters (p=0.7286). A statistically significant difference was observed for surgical time (310 min in transhiatal esophagectomy vs. 373 min in video-assisted thoracoscopy, p=0.0012) and anesthetic time (385 minutes in transhiatal vs. 467 min in video-assisted thoracoscopy, p<0.0001). A statistically significant difference was observed in the number of patients extubated at the end of the procedure (48.38% in transhiatal vs. 9.37% in video-assisted thoracoscopy, p=0.0022). Regarding gasometric parameters at the end of the surgical procedure, only pO_2_ showed a statistically significant difference (p=0.0010). Regarding ICU admission scores, there were no differences regarding APACHE II (p=0.6542), SOFA (p=0.8949), and SAPS III (p=0.7656).

**Conclusions:**

This study showed no differences between the transhiatal and thoracoscopic esophagectomy in the prone position, in prognostic score performance, studied operative parameters, ICU stay and hospital stay times, and perioperative mortality, in agreement with literature findings. The advent of minimally invasive techniques in video-assisted esophagectomies brought the same benefits as thoracotomy, offering greater safety in mediastinal dissection under direct vision, in addition to mitigating the physiological repercussions of thoracotomies.

## ARTICLE HIGHLIGHTS

Esophagectomy is still considered one of the most complex operations, due to the need for oncological radicality, being indicated after adequate and thorough preoperative evaluation

The main postoperative complications are cardiorespiratory and infectious

Patients needs in the immediate postoperative period, resources and support from Intensive Care Units (ICU) for their recovery, including ventilatory support, parenteral nutrition, and others.

The most commonly used severity scores in ICU are APACHE II, SOFA and SAPS III, which show the patient’s severity status or not, treatment effectiveness, and therefore guide decision-making.

## Summary

**CENTRAL MESSAGE:**

Esophagectomy is still considered one of the most complex operations due to the need for oncological radicality, being indicated after adequate and thorough preoperative evaluation and performed in institutions that have specialized medical teams and intensive care resources, considering its possible postoperative complications. It is performed by thoracotomy, transhiatal, and recently, with the progress of video surgery, by thoracoscopy in prone positions as a minimally invasive procedure. Considering esophagectomy being a major surgery, highly invasive, and of long duration, regardless of the access route or surgical tactic employed, in the immediate postoperative period, they need resources and support from intensive care units (ICUs) for their recovery, including ventilatory support, parenteral nutrition, and others. It is very important to employ in dices or scores to evaluate metabolic, hemodynamic, and infectious conditions that better guide the intensivist in their conduct and treatments.

**PERSPECTIVES:**

This study showed no differences between the transhiatal and tho racoscopic esophagectomy in the prone position in prognostic score performance, studied operative parameters, ICU stay and hospital stay times, and perioperative mortality, in agreement with literature findings. Transhiatal esophagectomy is a technique that emerged with the objective of reducing pulmonary morbidity caused by thoracotomy. Recently, the advent of minimally invasive techniques in video-assisted esophagectomies brought the same benefits as thoracotomy, offering greater safety in mediastinal dissection under direct vision and allowing more adequate lymphadenectomy, in addition to mitigating the physiological repercussions of thoracotomies.

## VISUAL ABSTRACT

**Figure:**
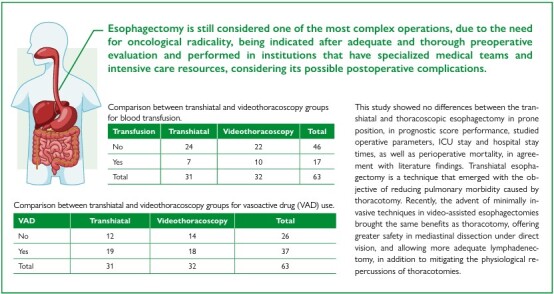

## RESUMO

**Racional:**

A esofagectomia é uma cirurgia de grande porte, invasiva e de longa duração; realizada em pacientes com comorbidades e condições nutricionais comprometidas. Os desafios históricos do tratamento cirúrgico do câncer esofágico são superar mortalidade, melhorar sobrevida e diminuir a morbidade.

**Objetivos:**

Comparar a morbidade transoperatória de duas técnicas cirúrgicas distintas de esofactomia no câncer esofágico, a esofagectomia transhiatal e a videotoracoscopia em posição prona, analisando parâmetros fisiológicos intraoperatórios, escores de admissão à Unidade de Terapia Intensiva (SOFA, SAPS III e APACHE II) e evolução pós-operatória.

**Métodos:**

Estudo retrospectivo, transversal, avaliando pacientes admitidos à Unidade de Terapia Intensiva (UTI) em pós-operatório imediato de esofagectomia em caráter eletivo por neoplasia de esôfago (carcinoma spinocelular e adenocarcinoma). Os dados foram obtidos de uma base de registro informatizado do Serviço de Terapia Intensiva e dos prontuários dos pacientes.

**Resultados:**

Foram avaliados 63 pacientes maiores de 18 anos de idade, distribuídos em dois grupos: 31 (49,21%) submetidos a esofagectomia transhiatal; e 32 (50,79%) submetidos a esofagectomia por videotoracoscopia. Não foi observado diferença estatisticamente significativa para tempo de internação em UTI (p=0,5309), tempo de internação pós-operatória (p=0,3066) e óbito no período perioperatório (30 dias, p=0,6562). Com relação aos parâmetros intraoperatórios, não foi observado diferença estatisticamente significativa para pacientes que receberam transfusão (p=0,2097); quantidade de hemoderivados em ml (p=0,2893); pacientes que fizeram uso de droga vasoativa (DVA) (p=0,9243); tempo de uso de DVA (p=0.9327); volume de líquidos infundidos em ml (p=0,7825); diurese em ml (p=0,7286). Observada diferença estatisticamente significativa para tempo cirúrgico (310 minutos na esofagectomia trans hiatal vs 373 minutos na videotoracoscopia, p=0,0012) e tempo anestésico (385 minutos na trans hiatal vs 467 minutos na videotoracoscopia, p<0,0001). Observada diferença estatisticamente significativa na quantidade de pacientes extubados ao final do procedimento (48,38% na transhiatal vs 9,37% na videotoracoscopia, p=0,0022). Com relação aos parâmetros gasométricos ao final do procedimento cirúrgico, apenas pO _2_ apresentou diferença estatisticamente significativa (p=0,0010). Com relação aos escores de admissão à UTI, não houve diferenças quanto ao APACHE II (p=0,6542), SOFA (p=0,8949) e SAPS III (p=0,7656).

**Conclusões:**

Este estudo não demonstrou diferenças entre a esofagectomia transhiatal e a toracoscópica em posição prona, no desempenho dos escores prognósticos, nos parâmetros operatórios estudados, nos tempos de internação na UTI e permanência hospitalar, bem como na mortalidade perioperatória, em concordância com os achados da literatura. O advento das técnicas minimamente invasivas nas esofagectomias videoassistidas trouxe os mesmos benefícios da toracotomia, oferecendo maior segurança na dissecção mediastinal sob visão direta, além de atenuar as repercussões fisiológicas das toracotomias.

## INTRODUCTION

 Esophagectomy is still considered one of the most complex operations due to the need for oncological radicality, being indicated after adequate and thorough preoperative evaluation and performed in institutions that have specialized medical teams and intensive care resources, considering its possible postoperative complications^5,9,36^. The main postoperative complications are cardiorespiratory and infectious. Publications from the 1990s and early 21st century show that hospital mortality up to the 30th day of esophagectomy can vary from approximately 2 to 7%, even when performed by experienced teams in this type of operation and in institutions with intensive care units (ICUs) adequate to receive and provide indispensable care to these patients in the immediate postoperative period^12,33^. General morbidity can vary from 35 to 50%, with respiratory complications being the most prevalent, ranging from 20 to 35%^6, 12,14, 16,25, 29,35^. 

 In addition to adequate preoperative evaluation of the patient’s clinical and nutritional conditions and the location and extent of the esophageal tumor or the esophagogastric junction, esophagectomy complications are also associated with the access route for esophageal removal and digestive tract reconstruction^8,10,19,23^. In the recent past, access routes for esophagectomy were transthoracic, with the Ivor-Lewis technique being widely used, or transhiatal. The most commonly employed digestive tract reconstruction uses a gastric tube constructed with the greater curvature of the stomach, with the esophagogastric anastomosis being performed inside the mediastinum or in the cervical region^ 1,8,9 ,46^. 

 Transhiatal esophagectomy, also called transmediastinal, described by Pinotti et al. and Orringer et al., was the surgery of choice for many years, employed by most services performing this operation for treating both adenocarcinoma of the esophagogastric junction and squamous cell carcinoma of the esophagus. In these procedures, the patient undergoes laparotomy or laparoscopy and left cervicotomy^17,28 ,42,43^ . 

 Recently, with the progress of video surgery and better equipment and instruments, the access route for esophagectomy began to be performed by videothoracoscopy. The best patient position option is lateral dorsal decubitus, also called the prone position. Thus, services that opt for this access route initially perform esophagectomy with the patient in the aforementioned position, then the patient is changed to the dorsal decubitus position and undergoes laparotomy and left cervicotomy, the gastric tube is constructed, and this tube is trans posed to the cervical region via the posterior mediastinum, and cervical esophagogastric anastomosis is performed^4,10,18 ,36,41 ,46^. 

 In summary, services that routinely practice esophagectomy for treating esophageal tumors can opt for the transhiatal route or videothoracoscopy. The choice of access route for esophagectomy may depend on the patient’s physical type, weight, clinical conditions, and tumor location, although in most cases, one or another access route can be employed^1,3,7,27,42 ,46^. 

 Therefore, esophagectomy, being a major surgery, highly invasive and of long duration, regardless of the access route or surgical tactic employed, patients most often have a history of smoking and alcoholism and present compromised nutritional conditions. Furthermore, intraoperatively, the patient may need infusion of many fluids, blood transfusions, and vasoactive drugs (VADs). Considering these arguments, in the immediate postoperative period, they need resources and support from ICUs for their recovery, including ventilatory support, parenteral nutrition, and others. It is very important to employ indices or scores to evaluate metabolic, hemodynamic, and infectious conditions that better guide the intensivist in their conduct and treatments^15,29,31 ,43,45^ . 

 The most commonly used severity scores in the ICU are APACHE II (Acute Physiology and Chronic Health Evaluation), SOFA (Sequential Organ Failure Assessment), and SAPS III (Simplified Acute Physiology Score), which show the patient’s severity status or not, treatment effectiveness, and therefore guide decision-making. Severity indices aim to provide a quantitative description of the degree of organic dys function of the patient, with their condition being translated into a numerical value, considering their clinical and laboratory conditions^13,15,31,34 ,40,44^ . 

 The objective of the present work is to analyze two groups of patients undergoing esophagectomy to treat esophageal cancer: transhiatal and videothoracoscopy, comparing the severity indices APACHE II, SOFA, and SAPS III in the immediate postoperative period and at ICU admission. 

## METHODS

 The study was retrospective, observational, cross-sectional, and quantitative, evaluating data obtained from a computerized registry database of the intensive care service of the Hospital de Clínicas of the State University of Campinas (UNICAMP). The computerized registry database was daily fed with data referring to clinical, physiological, laboratory, and score quantitative parameters by professionals from the ICU team^15^ . 

### Inclusion criteria

 The inclusion criteria for study participants were:

- Patients over 18 years old;
- Undergoing elective esophagectomy;
- Undergoing esophagectomy for neoplasia;
- Operated by the same team.

### Exclusion criteria

 The exclusion criteria were:

- Patients under 18 years old;
- Patients undergoing emergency surgery;
- Patients undergoing esophagectomy for non-neoplastic diseases;
- Patients undergoing esophagectomy for neoplasias other than adenocarcinoma or squamous cell carcinoma.

The database search retrieved 64 patients admitted in the immediate postoperative period to the ICU, undergoing elective esophagectomy for esophageal neoplasia, operated by the esophagus, stomach, and duodenum group, from January 2013 to October 2017. One patient operated for gastrointestinal stromal tumor in the esophagus was excluded. Participants were divided into two groups: transhiatal esophagectomy (31 patients) and videothoracoscopic esophagectomy (32 patients), operated with standardized operative techniques by a single surgical team. All patients remained postoperatively in a single ICU, therefore followed by a single team of intensivist physicians. 

### Transhiatal esophagectomy

 The surgical procedure begins with the patient in horizontal dorsal decubitus. Median laparotomy is performed; opening of the gastrocolic ligament and access to the retrocavity; section of short and left gastroepiploic vessels, with release of gastric fundus and posterior wall; lymphadenectomy of the gastric lesser curvature and celiac trunk; dissection of the diaphragmatic hiatus and anterior opening; left cervicotomy, with isolation and section of cervical esophagus; blunt manual dissection of thoracic esophagus through hiatal opening and cervicotomy; esophageal excision to the abdominal cavity; construction of gastric tube along lesser curvature, using mechanical staplers; transposition of gastric tube through hiatal opening to cervical region; manual end-to-side esophagogastric anastomosis. Intrathoracic and cervical lymph nodes found were removed. Jejunostomy for nutritional support were introduced and drainage of cervical anastomosis were performed^1,5,39 ,42^. 

### Videothoracoscopic esophagectomy

 The surgical procedure begins with the patient in the prone position. With orotracheal intubation with a wire-reinforced tube, with carbon dioxide pneumothorax, and cavitary pressure of 8 mmHg. The first 10 mm trocar was positioned in the fifth intercostal space at the midaxillary line, the second 5 mm in the seventh intercostal space at the posterior axillary line, and the third 10 mm in the fourth intercostal space at the posterior axillary line. Dissection was performed with atraumatic forceps for esophageal manipulation and monopolar electrocautery (hook), mobilizing the esophagus with its periesophageal lymphatic drainage chains and thoracic duct en bloc. Section of the azygos vein after ligatures with cotton thread and placement of metallic clips. Right and left paratracheal, superior and inferior tracheobronchial, infracarinal, periesophageal lymph node chains, and thoracic duct were removed en bloc. Finishing the thoracic time, a 28 French caliber drain was placed along the esophageal resection bed. Direct visualization of pulmonary re-expansion was performed. Then the patient was repositioned to dorsal decubitus, undergoing median laparotomy; opening of the gastrocolic ligament and access to the retrocavity; sectioning of the short and left gastroepiploic vessels, with release of the gastric fundus and posterior wall; lymphadenectomy of the gastric lesser curvature and celiac trunk; and dissection of the diaphragmatic hiatus. Left cervicotomy, with isolation and section of cervical esophagus; esophageal excision to abdominal cavity; construction of gastric tube through stapling along lesser curvature; exeresis of the surgical specimen; passage of gastric tube through hiatal opening to cervical region; manual end-to-side esophagogastric anastomosis. Jejunostomy for nutritional support were introduced and drainage of cervical anastomosis were performed^18,19,21,39^ . 

### Intraoperative parameters

 The following intraoperative parameters were evaluated:

- Surgical time (in minutes);
- Anesthetic time (in minutes);
- Need for blood transfusion and quantity (in ml);
- Need for VAD use and time of use (in minutes);
- Diuresis (in ml);
- Total volume of infused fluids (in ml);
- Last arterial blood gas of anesthetic time, collected after completion of the surgical act, with the following parameters evaluated:
- Partial pressure of oxygen (pO_2_);
- Partial pressure of carbon dioxide (pCO_2_);
- Bicarbonate anion concentration (HCO_3−_);
- Serum lactate level;
- Hemoglobin level (Hb);
- Hematocrit (Ht).
- Patient extubated at the end of the procedure (yes or no).

### Intensive care unit admission scores

 Prognostic scores for ICU admission were evaluated in the immediate postoperative period:

- SAPS III;
- APACHE II;
- SOFA.

The SAPS III score was calculated with data from the first hour of ICU admission, using the worst variables in this period. The APACHE II score was calculated with data from the first 24 h of admission, also using the worst variables in this period. The SOFA score is calculated with the worst result for each parameter. Additionally, the following parameters were evaluated during the hospital stay period:

- Days of postoperative hospitalization, obtained by subtraction between surgery day and hospital discharge day (or death);
- Days of ICU stay, obtained by subtraction between surgery day and clinical ICU discharge day;
- Deaths, considered during the entire hospital stay (in ICU and ward).

### Statistical analysis

 To describe the sample profile according to the variables under study, frequency tables of categorical variables with absolute frequency (n) and percentage (%) values were created, and descriptive statistics of numerical variables were calculated, with mean, standard deviation, minimum and maximum values, and median. The chi-square test was used for statistical comparison of categorical variables, and the Mann-Whitney test for numerical variables. For statistical analysis, the computer program the SAS System for Windows (Statistical Analysis System), version 9.4, SAS Institute Inc., 2002–2008, Cary, NC, USA, was used. The present study was approved by the Ethics and Research Committee of the Faculty of Medical Sciences (FCM) of UNICAMP (Nº 2.886.114). 

## RESULTS

 Sixty-three patients over 18 years old were evaluated who were admitted in immediate postoperative period of elective esophagectomy for neoplasia and operated from January 2013 to October 2017. Of the 63 patients, 31 (49.21%) underwent transhiatal esophagectomy, and 32 (50.79%) underwent esophagectomy with thoracic dissection by videothoracoscopy. There were 14 female patients (22.22%) and 49 male patients (77.78%). Comparing the transhiatal and videothoracoscopy subgroups, a statistically significant difference was observed (p=0.0127, p<0.05) (Table 1). 

**Table 1:** Comparison between transhiatal and videothoracoscopy groups according to sex.

Sex	Transhiatal	Videothoracoscopy	Total
Female	11	3	14
Male	20	29	49
Total	31	32	63

 The age range was 58.9±7.45 years. Comparing the two groups, a statistically significant difference was observed (p=0.0453, p<0.05) (Table 2). 

**Table 2:** Comparison between transhiatal and videothoracoscopy groups according to age range.

	Transhiatal	Videothoracoscopy	p-value
	Mean–Median (SD)	Mean–Median (SD)	
Age (years)	56.94–58 (7.84)	60.97–60.50 (6.58)	0.0453

SD: standard deviation.

 Twenty-five patients (39.68%) were operated for adenocarcinoma of the esophagogastric junction, and 38 patients (60.32%) for squamous cell carcinoma. Patients with squamous cell carcinoma were previously submitted to neoadjuvant radiotherapy and chemotherapy, and patients with adenocarcinoma were operated upfront1. Comparing the two groups, a statistically significant difference was observed (p=0.0090, p<0.05), adjusted for sex and age (Table 3). 

**Table 3:** Comparison between transhiatal and videothoracoscopy groups according to histological type.

Histological type	Transhiatal	Videothoracoscopy	Total
Adenocarcinoma	17	8	25
Squamous cell	14	24	38
Total	31	32	63

 Of the 63 patients analyzed, 46 (73.02%) did not receive blood products, and 17 (26.98%) received blood transfusions. Comparing the groups, no statistically significant difference was observed (p=0.2097, p>0.05), already adjusted for sex and age (Table 4). 

**Table 4:** Comparison between transhiatal and videothoracoscopy groups for blood transfusion.

Transfusion	Transhiatal	Videothoracoscopy	Total
No	24	22	46
Yes	7	10	17
Total	31	32	63

 Regarding the use of VAD, 26 patients (41.27%) did not use them during the surgical-anesthetic act, and 37 (58.37%) used them (Table 5). Comparing the two groups, no statistically significant difference was observed (p=0.9243, p>0.05), already adjusted for sex and age. 

**Table 5:** Comparison between transhiatal and videothoracoscopy groups for vasoactive drug use.

VAD	Transhiatal	Videothoracoscopy	Total
No	12	14	26
Yes	19	18	37
Total	31	32	63

VAD: vasoactive drug.

 At the end of the surgical procedure, 18 patients (28.57%) were extubated; however, 46 patients (71.43%) were sent to the ICU on mechanical ventilation. Comparing the two groups, a statistically significant difference was observed (p=0.0022, p<0.05), already adjusted for sex and age (Table 6). 

**Table 6:** Comparison between transhiatal and videothoracoscopy groups for extubation at the end of surgery.

Extubation	Transhiatal	Videothoracoscopy	Total
No	16	29	45
Yes	15	3	18
Total	31	32	63

 The quantitative variables of intraoperative parameters are summarized below, with comparison between subgroups already adjusted for sex and age (Table 7). 

**Table 7:** Comparison between transhiatal and videothoracoscopy groups for intraoperative parameters.

n=63	Transhiatal (n=31)	Videothoracoscopy (n=32)	p-value
	Mean±SD–Median (min–max)	Mean±SD–Median (min–max)	
Surgical time (min)	342.22±69.62–345(200–555)	310.32±79.20–290(200–555)	373.13±40.26–375(290–460)–0.0012
Anesthetic time (min)	427.46±82.22–435 (235–675)	385.65±86.15–375 (235–675)	467.97±53.80–467.50 (360–570) <0.0001
Blood transfusion (mL)	339.47±102.77–300 (200–600)	384±121.90–320 (280–600)	308.30±79.05–300 (200–500) 0.2893
VAD time (min)	172.57±93.14–170 (25–385)	171.32±76.39–175 (30–325)	173.89±110.39–155 (25–385) 0.9327
Fluid volume (mL)	5245.90±1329.89–5000 (1810–10500)	5184.27±1603.49–4900 (1810–10500)	5303.69±1033.31–5050 (3000–7500) 0.7825
Diuresis (mL)	631.56±369.06–500 (200–1700)	582.50±279.25–500 (200–1350)	679.03±438.52–500 (200–1700) 0.7286
pO_2_	163.33±43.65–155 (68–264)	144.44±36.65–142 (68–233)	180.44±42.89–177.50 (116–264) 0.0010
pCO_2_	37.88±4.78–36.90 (29.40–56.60)	38±5.34–37 (30.10–56.60)	37.77±4.29–36.90 (29.40–48.90) 0.6629
HCO_3_	21.80±2.02–21.30 (18.20–28.40)	21.76±2.20–21.40 (18.20–28.40)	21.84±1.93–21.20 (18.92–27.00) 0.8729
Lactate	2.15±1.15–1.90 (0.60–5.90)	2.23±1.08–2.05 (1–5.5)	2.16±1.23–1.85 (0.60–5.90) 0.6648
Hb	11.20±1.68–11 (7.40–15.90)	11.38±1.73–11.20 (9.20–15.90)	11.04±1.64–11 (7.4–15.1) 0.4127
Ht	34.54±5.06–34.10 (23.20–48.60)	35.08±5.20–34.70 (28.40–48.60)	34.06±4.96–34.10 (23.20–46.40) 0.4275

SD: standard deviation; min: minimum; max: maximum; VAD: vasoactive drugs; pO_2_: oxygen concentration; pCO_2_: carbon dioxide concentration; HCO_3_: bicarbonate; Hb: hemoglobin; Ht: hematocrit.

 The results of ICU admission scores (APACHE II, SOFA, SAPS III) are summarized in Table 8, with comparisons between subgroups already adjusted for sex and age. 

**Table 8:** Comparison between transhiatal and videothoracoscopy groups for prognostic scores at admission. There was no statistically significant difference between groups.

n=63	Transhiatal (n=31)	Videothoracoscopy (n=32)	p-value
	Mean±SD–Median (min–max)	Mean±SD–Median (min–max)	
APACHE II	9.63±2.97–10 (4–17)	9.03±2.69–8 (4–14)	10.22±3.16–10 (5–17) 0.6542
SOFA	3.21±±2.16–3 (0–8)	3.35±2.37–2 (0–8)	3.06±1.97–3 (0–7) 0.8949
SAPS III	40.98±7.12–40 (26–66)	39.67±6.57–39.50 (32–55)	42.22±7.49–42 (26–66) 0.7656

SD: standard deviation; APACHE II: Acute Physiology and Chronic Health Evaluation; SOFA: Sequential Organ Failure Assessment; SAPS III: Simplified Acute Physiology Score.

 The results regarding days of ICU stay and days of postop erative hospitalization are summarized below, with compari sons between groups already adjusted for sex and age (Table 9). 

**Table 9:** Comparison between transhiatal and videothoracoscopy groups for ICU stay time and postoperative hospitalization time.

n=63	Transhiatal (n=31)	Videothoracoscopy (n=32)	p-value
	Mean±SD–Median (min–max)	Mean±SD–Median (min–max)	
ICU stay (days)	6.9±7.14–6 (2–41)	5.84±2.35–6 (2–13)	7.94±9.72–5.50 (2–41) 0.5309
Postoperative hospitalization (days)	13.17±11.62–9 (2–65)	15.83±14.49–9 (4–65)	10.52±7.09–9 (2–34) 0.3066

SD: standard deviation; min: minimum; max: maximum; ICU: intensive care unit.

 There were five deaths (7.94%) among the 63 patients, two in the transhiatal group and three in the videothoracoscopy group (p=0.6562, p>0.05), already adjusted for sex and age. 

## DISCUSSION

 There are, in clinical practice in intensive care, two main categories of scores: SOFA, which synthesizes physiological de rangements by organs and provides an objective assessment of the extent and severity of dysfunction. It comprises six organ systems, with 0 to 4 points assigned according to the degree of dysfunction, also based on laboratory tests. Although it was created to describe morbidity, a correlation between SOFA score and mortality was observed through retrospective analy sis of the European/North American Study of Severity System database, which indicated good correlation of the score with survival, as well as good distribution of patients among differ ent score values^15 ,34^.

 Daily scores can be calculated and used to describe the de gree of organ dysfunction during a patient’s ICU stay, in contrast to prognostic systems designed to give a prediction based mainly on the time of ICU admission^15,44^. The mean found in the research was 3.21; for the transhiatal group, it was 3.35, and in the videothoracoscopy group, it was 3.06. This differ ence was not statistically significant (p=0.8949, p>0.05).

 The other main category is the disease severity prognos tic model, evaluated by APACHE and SAPS, which employs vital physiological data and information related to the nature of the current disease at the time of ICU admission to predict the probability of death. The APACHE II score was presented more than 35 years ago, in 1985, using data from 5,815 pa tients hospitalized between 1979 and 1982 in 13 hospitals in the United States. It was developed to estimate disease severity and predict hospital mortality, both for deaths occurring in the ICU and for those occurring in wards after ICU discharge. For its calculation, the worst values of laboratory tests in the first 24 h of ICU hospitalization, age, and chronic diseases pri or to ICU admission should be considered^15 ,24,31 ,34,40^. The mean found in the research was 9.63; in the transhiatal group it was 9.03; and in the videothoracoscopy group it was 10.22, with no statistically significant difference (p=0.6542, p>0.05).

 The SAPS III score was developed using data from 303 ICUs and 16,784 patients. It is composed of 20 different variables of laboratory tests and vital data, easily measurable in the first hour of patient admission to the ICU. For each of the analyzed variables, a weight is assigned according to the severity of the physiological disturbance. Theoretically, the lowest value attributed by the score is 16, and the highest is 217 points. The physiological variables that compose the acute physiological score are temperature, systolic blood pressure, heart and respiratory rate, blood oxygenation, arterial pH, sodium, potassium, creatinine, bilirubin, hematocrit, leukocytes, platelets, and Glasgow coma scale^31,34 ,40^.

 However, the SAPS III system was not developed to be representative of all types of patients, especially in specific ar eas or individual types of diseases, as it was designed for the general ICU population. The SAPS III model prediction is based exclusively on data evaluated within the first hour of ICU admission^34^ . The general mean of the sample was 40.98; in the transhiatal group, the mean was 39.67, and in the vid eothoracoscopy group, it was 42.22.

 The consulted literature is poor in comparing the perfor mance of prognostic scores of patients undergoing two distinct surgical techniques for esophagectomy, transhiatal and video thoracoscopy. In this research, no statistically significant differences were observed between the two groups when comparing prognostic scores. 

 The physiological variables of the three scores are composed of laboratory data and vital signs. Creatinine, a variable used in all three scores, changes above normal limits only when glomerular function is below 50%; diuresis is affected in even later stages of renal failure^13,15^. The diuresis observed intraoperatively also showed no statistically significant difference (p=0.7286, p>0.05). Platelet count is part of SOFA and SAPS III. Postoperative thrombocytopenia occurs due to decreased production, increased platelet destruction, sequestration, or dilution^13,40,44^. Dilution generated by volume replacement or intense blood transfusion would be mechanisms that could affect platelet count in the immediate postoperative period, reflecting on admission scores. However, both fluid replacement (mean of 5184.27 ml in transhiatal esophagectomy and mean of 5303.69 ml in videothoracoscopy, p=0.7825, p>0.05) and blood product volume (384 ml in transhiatal and 308.30ml in video-thoracic assisted (p=0.2893, p>0.05)) also showed no significant differences. Bilirubin dosage, a variable present in SOFA and SAPS III, may be increased in the context of non-hepatobiliary surgeries as a consequence of hematoma reabsorption, polytransfusion, hepatocellular lesions, resulting from ischemia, and drug hepatitis (sulfonamides, phenytoin, and halogenated anesthetics); however, in the context of elective abdominal surgery, such effects are rare (less than 1%). Similarly, leukocyte count, a variable present in SAPS III and APACHE II, tends to vary similarly for both groups, with elevation in the immediate postoperative period and subsequent gradual decline. The pH value, present in APACHE II and SAPS III, as well as sodium, potassium, and hematocrit values, present only in APACHE II, reflect the anesthetic care and intensivist team^13 ,22,31 ,40,44^ . 

 Unlike other parameters, the Glasgow coma scale score to be used is the best value in APACHE II. In patients using sedatives, it is considered normal, thus disregarding the extubation difference between subgroups^24^ . For SOFA calculation, in sedated patients, the score corresponding to the value the patient would receive if not sedated is attributed. In SAPS III, for patients with tracheal intubation, the best score is attributed in the verbal response assessment if they did not have neurological problems, with a score of 1 considered when ocular and motor responses are evaluated according to the Glasgow scale^40^ . 

 This study did not record a statistically significant difference regarding the number of patients who used VADs intraoperatively. The age difference between patients undergoing the two esophagectomy techniques did not affect the scores, since both are within the same age scoring range. Additionally, patients admitted for elective surgery receive the same scoring value if there are chronic conditions. The comorbidity profile of the two subgroups is similar, considering that patients with important comorbidities are not candidates for esophagectomy^12,14 ,16^. 

 The cardiorespiratory repercussions described in the introduction are greater in patients undergoing thoracotomy in left lateral decubitus, and selective ventilation. Even such alterations are reversed intraoperatively with reestablishment of ventilation in both lungs. Pressure lability and arrhythmias that occur during mediastinal manipulation in transhiatal esophagectomy are also reversed as manipulations cease^6,9,14,16,33^. The two surgical techniques discussed in this research involved the use of nonselective ventilation, which would help explain the absence of differences in respiratory parameters of the scores. 

 A study conducted by Yatabe et al., evaluating a sample of 15 patients undergoing minimally invasive esophagectomy (cervical lymphadenectomy, thoracoscopy, and laparoscopy), with thoracic dissection in the prone position and selective ventilation, observed an APACHE II admission score of 13±4 and a SOFA score of 2±1. The sample mean age was 66±10 years, which could justify the higher APACHE II score. The surgical (605 min) and anesthetic (668 min) times described were longer than those found in this research. However, the surgical technique employed by the authors was totally minimally invasive^45^ . 

 Mitaka et al.^32^ analyzed 40 patients undergoing esophagectomy by right thoracotomy and laparotomy, with a mean age of 66 years, and observed that the APACHE II admission score was 10 and SOFA was 3. 

 Arméstar et al.^2^ evaluated 159 patients undergoing esophagectomy by right thoracotomy and laparotomy with selective ventilation and left lateral decubitus, with a mean age of 58 years, and observed an APACHE II score of 12.1. 

 Park et al., analyzing 7227 patients admitted to ICUs between 1995 and 2007 in the United Kingdom after elective esophagectomy for cancer, recorded that the mean APACHE II score was 13.8, with a population with a mean age of 64 years^37^ . 

 The longer surgical time in the videothoracoscopy group (mean of 373.13 min) and anesthetic time (mean of 467.97 min), compared to the transhiatal group (310.32 and 385.65 min, respectively) determined the lower extubation rate: 9.37% in the videothoracoscopy group versus 51.16% in the transhiatal group, extubated at the end of the procedure by the anesthetic team (p=0.0022, p<0.05). This fact was also reflected in the pO_2_ values of blood gas analysis at the end of the surgical procedure (144.44 in transhiatal, and 180.44 in videothoracoscopy, p=0.0010, p<0.05). 

 Compared to transhiatal esophagectomy, videothoracoscopy adds important operative time due to patient positioning, decubitus change, and meticulous mediastinal dissection. Comparing transhiatal dissection with thoracotomy, Márton et al., in a retrospective study, observed longer operative time in thoracotomy; however, without differences regarding transfusion need and mortality. Also, they observed no differences regarding serum procalcitonin dosage and microalbuminuria or inflammatory response markers in the early postoperative period^30^ . 

 Schoppmann et al. retrospectively compared 31 patients undergoing videothoracoscopic esophagectomy in left lateral decubitus with 31 patients undergoing thoracotomy, with selective ventilation in both groups. They observed fewer transfused patients, a lower incidence of pneumonia, shorter mechanical ventilation time, and shorter ICU and hospital stay times in the videothoracoscopy group^38^ . 

 Kanekiyo et al. evaluated 85 patients undergoing videothoracoscopic esophagectomy in the prone position and selective ventilation, with abdominal time by videolaparoscopy, and 104 patients undergoing esophagectomy by thoracotomy in the left lateral decubitus and laparotomy. They observed longer surgical time, less bleeding, shorter postoperative stay, and fewer pulmonary complications in videothoracoscopy. Disease-free survival at 5 years and overall survival showed no statistically significant differences^21^ . 

 Li et al. made a retrospective analysis of 38 patients undergoing videothoracoscopic esophagectomy in the left lateral decubitus and selective ventilation, comparing them with 21 patients undergoing videothoracoscopy in the prone position, with non-selective ventilation and pneumothorax. No differences were observed regarding blood loss, intraoperative respiratory parameters, or postoperative pain scores. Thoracoscopy in the prone position showed shorter thoracic surgical time and shorter ICU stay^26^ . 

 Jínek et al.^20^ and Deo et al.^11^ compared patients undergoing hybrid videothoracoscopic esophagectomy with patients undergoing transhiatal esophagectomy, observing longer surgical time in videothoracoscopy. No significant differences were observed for blood loss and postoperative hospital stay. In our study, there were no statistically significant differences regarding ICU stay time (p=0.5309, p>0.05) and postoperative hospital stay (p=0.3066, p>0.05). Additionally, no differences were observed regarding mortality (p=0.6562, p>0.05). 

## CONCLUSIONS

 The study showed no differences between the two studied groups in prognostic score performance, studied operative parameters, ICU stay and hospital stay times, and perioperative mortality, in agreement with literature findings. Transhiatal esophagectomy is a technique that emerged with the objective of reducing pulmonary morbidity caused by thoracotomy. Recently, the advent of minimally invasive techniques in video-assisted esophagectomies brought the same benefits as thoracotomy, offering greater safety in mediastinal dissection under direct vision and allowing more adequate lymphadenectomy, in addition to mitigating the physiological repercussions of thoracotomies. 

## Data Availability

The Information regarding the investigation, methodology, and data analysis of the article are archived under the responsibility of the authors.
